# Antifungal activity of silver nanoparticles prepared using *Aloe vera* extract against *Candida albicans*

**DOI:** 10.14202/vetworld.2023.18-26

**Published:** 2023-01-06

**Authors:** Mbarga Manga Joseph Arsène, Podoprigora Irina Viktorovna, Marukhlenko Alla, Morozova Mariya, Senyagin Alexander Nikolaevitch, Anyutoulou Kitio Linda Davares, Mumrova Evgenia Yurievna, Manar Rehailia, Ada Arsene Gabin, Kulikova A. Alekseevna, Yashina Natalia Vyacheslavovna, Zhigunova Anna Vladimirovna, Orlova Svetlana, Das Milana

**Affiliations:** 1 Department of Microbiology V.S. Kiktenko, Medical Institute, Peoples Friendship University of Russia (RUDN University), Moscow, Russia; 2 Research Institute of Molecular and Cellular Medicine, Peoples Friendship University of Russia (RUDN University), Moscow, Russia; 3 Department of Pharmaceutical and Toxicological Chemistry, Medical Institute, Peoples Friendship University of Russia (RUDN University), Moscow, Russia; 4 Department of Agrobiotechnology, Agrarian Institute, Peoples Friendship University of Russia (RUDN University), Moscow, Russia; 5 Department of Traumatology and Orthopedics, Medical Institute, Peoples Friendship University of Russia (RUDN University), Moscow, Russia; 6 Department of Oral and Maxillofacial Surgery, Medical Institute, Peoples Friendship University of Russia (RUDN University), Moscow, Russia; 7 Department of Dietetics and Clinical Nutritiology, Medical Institute, Peoples Friendship University of Russia (RUDN University), Moscow, Russia

**Keywords:** *Aloe vera*, antifungal, antimicrobials, green synthesis, silver nanoparticles

## Abstract

**Background and Aim:**

Resistance to antifungal agents is a serious public health concern that has not been investigated enough because most studies on antimicrobials are dedicated to antibacterial resistance. This study aimed to synthesize silver nanoparticles (AgNPs) using *Aloe vera* extract, and to assess its antifungal activity against *Candida albicans*.

**Materials and Methods:**

Silver nanoparticles were synthesized by reducing Ag nitrate with aqueous *A. vera* extracts. Physicochemical properties of synthesized AgNPs were determined by ultraviolet–visible spectrophotometry, photon cross-correlation spectroscopy, energy-dispersive X-ray fluorescence spectrometry, X-ray diffraction analysis, and Fourier-transform infrared spectroscopy. An antifungal investigation was performed against four clinical *C. albicans* (C1, C2, C3, and C4) and a reference strain, *C. albicans* ATCC 10321.

**Results:**

Cubic AgNPs with a mean X50 hydrodynamic diameter of 80.31 ± 10.03 nm were successfully synthesized. These AgNPs exhibited maximum absorbance at 429.83 nm, and X-ray fluorescence (XRF) confirmed the presence of Ag in AgNPs solution by a characteristic peak in the spectrum at the Ag Kα line of 22.105 keV. Infrared spectra for AgNPs and *A. vera* extract indicated that the compounds present in the extract play an essential role in the coating/capping of synthesized AgNPs. Different concentrations (200, 100, 50, 25, 10, and 5 μg/mL) of AgNPs were tested. The antifungal activity was shown to be dose-dependent with inhibition zones ranging from 10 mm to 22 mm against *C. albicans* ATCC 10231, 0 mm to 15 mm against C1, 0 mm to 16 mm against C2 and C3, and 0 mm to 14 mm for C4. Minimum inhibitory concentration ranged from 16 μg/mL to 32 μg/mL against clinical *C. albicans* (C1, C2, C3, and C4) and was 4 μg/mL against *C. albicans* ATCC 10231.

**Conclusion:**

This study showed the ability of *A. vera* to serve as an efficient reducing agent for the biogenic synthesis of AgNPs with excellent antifungal activity.

## Introduction

Antifungal resistance is a significant public health concern that receives less attention as it is overshadowed by the emergence of antibiotic resistance in bacteria [1, 2]. In recent years, several researchers have reported limitations to antifungal treatments, especially regarding their toxicity and growing resistance to antifungal agents [1–5]. The toxicity of most available effective antifungal agents is based on polyenes (amphotericin B), triazoles (fluconazole, itraconazole, voriconazole, and posaconazole), or echinocandins (caspofungin, micafungin, and anidulafungin), whose administration is accompanied by direct toxicity or adverse effects including toxicity and drug interactions [1, 6]. Moreover, antifungal resistance has been increasing worldwide [1, 7]. This resistance complicates treatment, induces more frequent outpatient visits and hospitalizations, and increases the cost of treatment. It has been reported that some resistant fungal species, like *Candida albicans*, responsible for fungal infections, including sepsis, are associated with mortality rates of up to 40% [1]. Due to that, other options for effective antifungal therapy must be illustrated to avoid the above-mentioned adverse effects.

Recently, interest in metallic nanostructures and nanocomplexes has increased considerably. Panáček *et al*. [1] reported that the strong bactericidal activity of silver (Ag) nanoparticles (AgNPs) against both Gram-positive and Gram-negative bacteria, including multiresistant strains, makes them a potential antifungal agent [8]. The synthesis of AgNPs is performed in physical ways (evaporation-condensation and laser ablation) [9] or by chemical reduction using inorganic and organic reducing agents, such as poly (ethylene glycol), sodium borohydride, N-dimethylformamide, hydrazine, and surfactant template approach [10, 11]. Although these synthesis routes are effective, they cause toxicity, and more reliable methods need to be developed [8, 9]. More eco-friendly methods, such as green routes using microorganisms, enzymes, and plant extracts, are increasingly suggested to replace chemical methods [10, 12–16]. The biogenic synthesized AgNPs were more stable and cost-effective [9]. Several plants, medicinal or not, such as *Azadirachta indica* [10, 11], *Citrus medica*, *Tagetes lemmonii*, *Tarenna asiatica* [14], *Rosa canina* [17], and *Syzygium cumini* [16] have already been used to synthesize and stabilize metallic biogenic AgNPs. A thorough literature survey indicated that very few studies have been performed using *Aloe vera* in nanotechnology [18].

**Figure-1:**
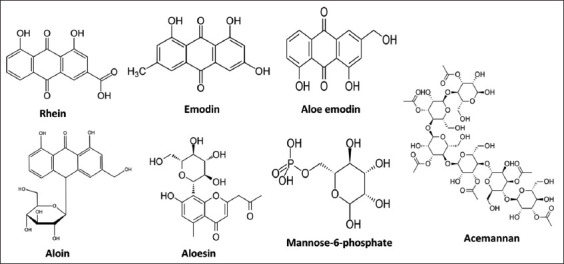
Phytochemical constituents of *Aloe vera*.

*Aloe vera* is a plant of the Liliaceae family whose medicinal properties have been known since ancient times. Collenchyma and thin-walled cells from the parenchyma of its leaves contain mucilaginous transparent gel, referred to as *A. vera* gel [19]. *Aloe vera* gel, extracts, juice, and powder have become popular and generally recognized as safe substances for applications in food, dietary supplements, and Ayurvedic drugs [19]. Several studies have demonstrated its anti-inflammatory, antibacterial, antioxidant, antiviral, anti-ulcer, wound healing, lipid-lowering, antidiabetic, antihypertensive, and immune regulator properties [19–22]. Studies on its chemical composition revealed that the main active phytoconstituents of *A. vera* are aloin, emodin, rhein, acemannan, *Aloe emodin*, mannose-6-phosphate, and aloesin [19], whose structures are depicted in Figure-1. Since *A. vera* is known and regarded as a safe product, molecules from its extract that may persist as capping and stabilizing elements on the surface of AgNPs would not constitute a toxicity concern [23].

This study aimed to synthesize AgNPs using *Aloe ver*a extract and to assess its antifungal activity against *Candida albican*s.

## Materials and Methods

### Ethical approval

Ethical approval was not required for this study. All the experiments were performed *in vitro*.

### Study period and location

This study was conducted from January 2022 to May 2022 at the Laboratory of Microbiology and Virology of the Medical Institute of the People’s Friendship University of Russia, Moscow, Russia.

### Plant collection

*Aloe vera* was collected in December 2021 from Nlobison II, Central Cameroon (VJ5J+C3 Yaounde, Cameroon) and identified using the mobile professional version of PictureThis - Plant Identifier App (Glority LLC, 2021). The plants were packaged in airtight plastic bags and transported to the microbiology laboratory of RUDN University on January 5, 2022.

### Extract preparation

Ten grams of *A. vera* leaves were weighed and added to 90 mL of distilled water in a conical flask, covered tightly, and shaken at 10× *g* for 2 h at 25°C in a shaker incubator (Heidolph Incubator 1000 coupled with Heidolph Unimax 1010, Germany). The mixture was then filtered using Whatman filter paper No. 1 [24].

### Phytofabrication of AgNPs

The aqueous extract of *A. vera* (20 mL) was added to 180 mL of 1 mM silver nitrate (AgNO_3_) solution (PanReac AppliChem), shaken at 4× *g* in a shaker incubator (Heidolph Incubator 1000 coupled with Heidolph Unimax 1010) in the dark for 24 h at 37°C for bioreduction process. The color change from colorless to brownish indicated the formation of AgNPs by bioreduction of Ag^+^ ions to Ag^0^ [24].

### Characterization of phytofabricated AgNPs

The first step in characterization was a visual observation. The color change indicated the reduction of Ag^+^ ions to Ag^0^ nanoparticles. We further analyzed the AgNPs with ultraviolet–visible (UV–Vis) spectrophotometry, photon cross-correlation spectroscopy, energy-dispersive XRF spectrometry, X-ray diffraction (XRD) analysis, and Fourier-transform infrared (FTIR) spectroscopy.

Ultraviolet–visible spectra were recorded with PerkinElmer Lambda 950 spectrophotometer (PerkinElmer, Inc., Waltham, USA) from 350 nm to 800 nm at a resolution of 1 nm, and the AgNO_3_ solution extract free was used as blank.

Particle hydrodynamic size and distribution were investigated with a Nanophox instrument with UVette cuvettes (routine pack, Sympatec GmbH), measurements were integrated to produce a single distribution with the photon cross-correlation spectroscopy (PCCS) Windox 5 software, and the size distributions were obtained using the non-negative least squares algorithm. Standard latex samples (20 ± 2 nm) (Sympatec GmbH, Clausthal-Zellerfeld, Germany) and blank samples were analyzed before the measurements to ensure the high accuracy of the measurements.

An EDX-7000 Shimadzu energy-dispersive XRF spectrometer (Japan) was used with the following settings: Range of measured elements – 11Na – 92U; X-ray generator a tube with an Rh-anode, air-cooled; voltage 4 kV–50 kV and current 1 μA–1000 μA; irradiated area a circle of 10 mm in diameter; silicon (Si) drift detector, counting method a digital counting filter; the content of elements according to the value of intensity; automatic change of filters emitting the wavelengths of the corresponding elements; and chamber size 300 mm × 275 mm × 100 mm. The XRF spectrum for each measurement was recorded at the same device settings: Mylar film, collimator width – 10 mm, exposure time – 100 s, atmosphere – air, and the number of repeated measurements for one sample n = 3. To process the obtained results, we used the OriginPro 2017 software (Version 94E, Origin-Lab Co., Northampton, MA, USA). The results obtained using the XRF method are presented as values of irradiation intensity expressed in cps/μA.

X-ray diffraction pattern was obtained by Bruker d8 advance using 1.541A (Cu-Ka). Fourier-transform infrared measurements were performed on AgNPs and *A. vera* crude-dried extract using Agilent Cary 630 FTIR spectrophotometer with a diamond ATR accessory (Agilent Technologies, USA). The spectral range was 4000–750 cm^−1^. The resolution was <2 cm^−1^, the correctness of the wavenumber was 0.05 cm^−1^, and the reproducibility of the wavenumber was 0.005 cm^−1^. The thickness of the absorbing layer was 1.5 nm (the clamping device guarantees the setting of optimal and reproducible pressure). The standard Agilent MicroLab Expert software (Agilent Technologies, Palo Alto, CA, USA) was used to control the device, measure the data, and evaluate the quality of the obtained spectra. The FTIR spectra were visualized in the wavenumber in the coordinates, cm^−1^ transmission, and %. All subsequent mathematical transformations of the spectral data array were performed using the OriginPro 2017 software (Version 94E, Origin-Lab Co., Northampton, MA, USA).

### Antimicrobial assessment

#### Preparation of antifungal solution

The solution containing green synthesized AgNPs was centrifuged at 21428× *g* for 1 h. The pellet was recovered, washed twice with ethanol, three times with distilled water, dried, and diluted to obtain a concentration of 1024 μg/mL. The stock solution was used to prepare the different concentrations used in the analytical process and was sterilized by microfiltration (0.22 μm) (Merck Millipore, Tullagreen, Cork, Ireland) before use [24].

#### Fungal culture

Four clinical strains of *C. albicans* (C1, C2, C3, and C4) and a reference strain *C. albicans* ATCC 10231 were used for this study. All these microorganisms were kindly provided by the Department of Microbiology V.S. Kiktenko of RUDN University. The different strains were cultured at 37°C for 48 h in 10 mL of SDB (Sabouraud Dextrose Broth) (HiMedia™ Laboratories Pvt. Ltd., India). After incubation, the culture medium containing the cells was centrifuged (7000× *g*, 4°C, 10 min), washed twice with sterile saline, and aseptically prepared in 5 mL of sterile saline to achieve a concentration equivalent to McFarland 0.5 using DEN-1 McFarland Densitometer (Grant-bio, Grant instruments Ltd., Cambridge, UK) [24].

#### Antifungal tests

The antifungal activity was assessed by the well diffusion method (inhibition diameters) and microbroth dilution method (for minimum inhibitory concentrations [MICs] and minimum fungicidal concentration [MFC]) [2].

The well diffusion method was used as described in our previous study [2] without any modification. After pouring 15 mL of sterile Sabouraud Dextrose Agar (SDA HiMedia™ Laboratories Pvt. Ltd.) into Petri dishes, 100 μL of each microorganism was spread. Then, AgNPs (200, 100, 50, 25, 10, and 5 μg/mL) were introduced into 20 μL wells. All the trials were performed in triplicate, the sterile distilled water used to dilute the AgNPs was used as a negative control. After incubation at 37°C for 24 h, the inhibition diameters were measured.

Additionally, MIC is the lowest concentration of antifungal agent that completely inhibits fungal growth, while MFC is the lowest concentration that kills 99.99% of the microorganism tested. The MIC and MFC of AgNPs were assessed using the microbroth dilution method in sterile U-bottom 96-well microplates as described by Arsène *et al*. [24] without any modification. Briefly, a serial 2-fold dilution of the AgNPs in SDB was performed in sterile U-bottom 96-well microplates. A 100 μL of broth was added to all the wells of the plates and 100 μL of AgNPs (1,024 μg/mL) was added to the first row. Serial dilutions were performed by transferring 100 μL of the solution. For each test well, 10 μL of the respective inoculum was added (with turbidity equivalent to a 0.5 McFarland scale). Finally, the plates were covered and incubated at 37°C for 24 h. After incubation, MIC was considered the lowest concentration of the tested material that inhibited the visible growth of the fungi. The MFCs were determined by subculturing the wells without visible growth (with concentrations ≥MIC) on SDA plates. Inoculated agar plates were incubated at 37°C for 24 h. The MFC was considered the lowest concentration that did not yield any bacterial growth on agar.

As described previously by Arsène *et al*. [24], the tolerance level of the tested bacterial strains against AgNPs was determined using the following formula: Tolerance = MBC/MIC; and interpreted as follows: MBC/MIC ≥16, the antifungal efficacy is considered as fungistatic whereas MBC/MIC ≤4 indicates fungicidal activity.

## Results and Discussion

### Biosynthesis and characterization of AgNPs

**Figure-2:**
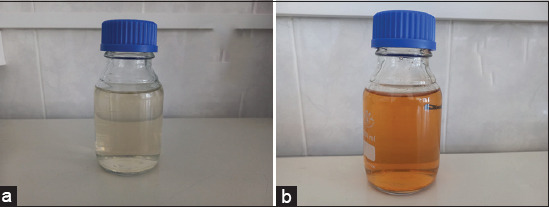
Coloration of solution before (a) and after (b) the phytofabrication process of silver nanoparticles with *Aloe vera* extract.

The first step in the characterization of AgNPs is visual observation. As shown in Figure-2, adding *A. vera* extract to 1 mM AgNO_3_ solution in darkness changed its color to brownish, indicating the formation of AgNPs. It has been reported that this color change is due to the excitation of surface plasmon vibrations in AgNPs [25, 26]. The result is not presented here, but it is worth mentioning that in this study, only the concentration of 1 mM was used after preliminary tests because higher concentrations (>2 mM) automatically led to the formation of blackish precipitates at the bottom of the solution. Othman *et al*. [27] reported the same observation for AgNO_3_ concentrations above 1.5 mM without explaining. In a recent study, we investigated the effect of AgNO_3_ concentration on the size of AgNPs. We found that high AgNO_3_ concentrations resulted in large particle sizes (>300 nm) [24], which could explain the precipitation of the particles.

**Figure-3:**
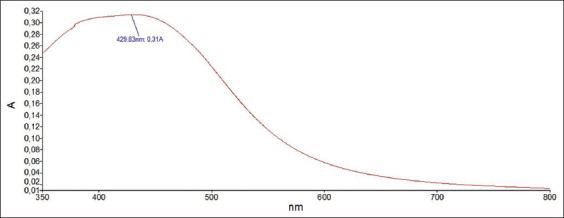
Ultraviolet–visible spectra of phytofabricated silver nanoparticles using *Aloe vera* extract.

**Figure-4:**
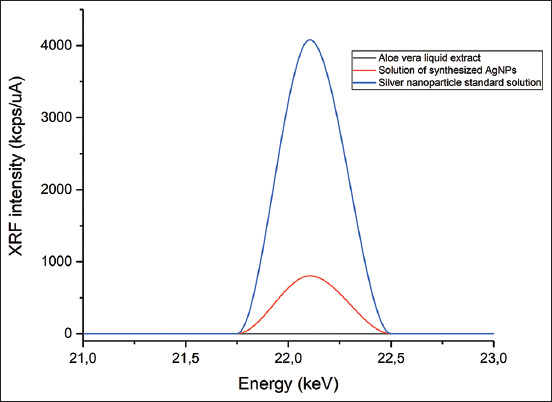
XRF spectra of liquid *Aloe vera* plant extract (black), solution of green synthesized AgNPs (red), and AgNP standard (blue) at the characteristic energy of silver fluorescence – silver Kα line of 22.105 keV. XRF=X-ray fluorescence, AgNPs=Silver nanoparticles.

**Figure-5:**
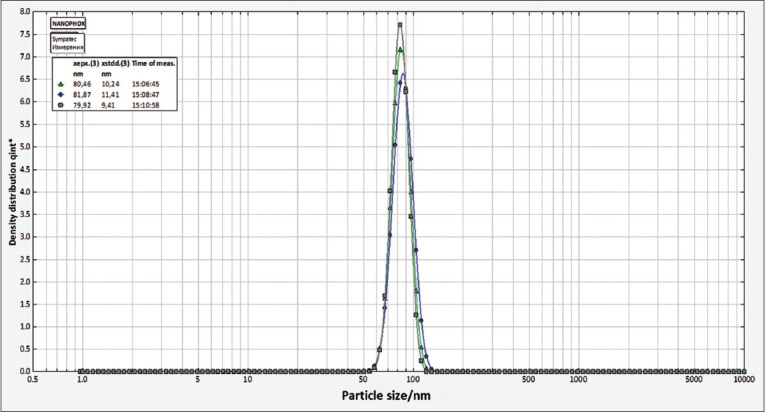
Particle size of phytofabricated silver nanoparticles using *Aloe vera* extract.

**Figure-6:**
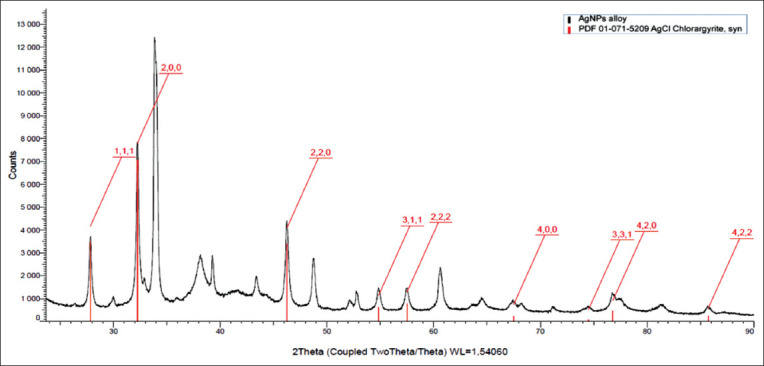
XRD pattern of phytofabricated silver nanoparticles using *Aloe vera* extract. XRD=X-ray diffraction.

**Figure-7:**
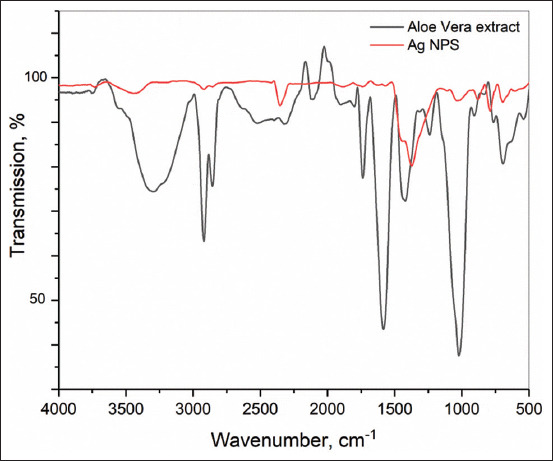
FTIR spectra of *Aloe vera* dried extract and green synthesized AgNPs. FTIR=Fourier transform infrared, AgNPs=Silver nanoparticles.

After visual observation, the AgNPs were further characterized by UV–Vis spectrophotometry, PCCS, XRD analysis, energy-dispersive XRF spectrometry, and FTIR spectroscopy. The UV–visible spectra of *A. vera*-mediated AgNPs showed maximum absorption peak bands at 411.6 nm (Figure-3). Similar results of wavelength were obtained for cauliflower (*Brassica oleracea*) and orange (*Citrus sinensis*) peel extract-mediated synthesis of AgNPs [28, 29]. The AgNPs are generally known to exhibit a UV-visible absorption maximum of 400–500 nm because of surface plasmon resonance [30]. Furthermore, XRF analysis confirmed the presence of Ag in the solution of AgNPs by a characteristic peak in the spectrum at the Ag Kα line of 22.105 keV (Figure-4). It is important to highlight that instead of the reaction mixture (*A. vera* extract + AgNO_3_), the purified and dried AgNPs were resuspended in distilled water for XRF analysis because the Ag^+^ ions of the reaction mixture could have biased the result. Indeed, it is well known that XRF analysis cannot distinguish ions of the same element in different valence states and detects all elements regardless of the form (here, Ag or Ag^+^) in which they are in the solution [31]. Furthermore, as depicted in Table-1, the XRF made it possible to establish differences in the elemental composition of *A. vera* extract and AgNPs solution. The Si, chlorine (Cl), and potassium (K) were found in both samples, but some elements that were present in the extract (sulfur and bromine) were not found in the AgNPs solution. The Ag was found in the AgNPs solution but not in the extract, thus confirming the formation of AgNPs. Interestingly, the mean fluorescence intensity of Ag (0.8046 cps/uA) in the AgNPs solution was at least 10 times greater than the other elements (Table-2), indicating that Ag was the most abundant element in the solution. Moreover, the PCCS revealed that the X50 hydrodynamic diameter of AgNPs was 80.31 ± 10.03 nm (Figure-5). Given that the AgNPs are defined as particles having a diameter ranging from 0 nm to 100 nm [24], and given the results presented above, it could be easily concluded that *A. vera* extract was successfully used to synthesize AgNPs in this study. Otherwise, the crystalline nature of AgNPs was confirmed by XRD analysis, and the XRD pattern revealed Bragg’s reflections that represent the face-centered cubic structure of Ag. In Figure-6, the XRD pattern presents diffraction peaks at 27.90, 32.23, 46.29, and 54.91, corresponding to lattice plane values recorded at (111), (200), (220), and (311) of Ag crystals, respectively. The obtained peaks in the XRD pattern clearly explained that the Ag^+^ ions had been completely reduced to Ag^0^ by the reducing and stabilization compounds in the aqueous extract under reaction conditions [32]. There were some unassigned peaks in the chromatogram. The presence of sharp peaks in the chromatogram indicated proteins or some bioorganic compounds in the NPs during synthesis [32]. Similar results of crystalline were reported by Rudrappa *et al*. [32] on *Plumeria alba*-mediated AgNPs with XRD spectrum pattern that showed distinct diffraction peaks at 2q angles of 38.19°, 44.37°, 64.77°, and 76.39°, indexed to the (111), (200), (220), and (311) Bragg’s reflection of the face-centered cubic structure of Ag crystals, confirming their highly crystalline nature [32]. In another work by Krishna *et al*. [33], AgNPs from a leaf extract of *Sansevieria roxburghiana* showed peaked values at 38°, 44°, 64°, and 77°, confirming the crystalline face-centered cubic nature. In addition, FTIR analysis was carried out to identify possible biomolecules present in *A. vera* extract responsible for reducing Ag^+^ into Ag^0^. Figure-7 presents the FTIR spectra of phytofabricated AgNPs and *A. vera* extract. The broadband between 3200 cm^−1^ and 3500 cm^−1^ on *A. vera* extract spectrum corresponds to the N-H stretching of primary aliphatic amines or can be substantiated by vibrations of the O-H bond in alcohols and phenols (Figure-7). Peaks at 2916, 2860, and 1587 cm^−1^ correspond to stretching vibrations of secondary amines or their salts (NH_3_^+^). The assignments at 2121 and 1795 cm^−1^ correspond to double bonds of the aromatic frame. Absorption peaks at 1732 cm^−1^ are due to C=O from aldehydes. Peaks at 1245 and 1016 cm^−1^ correspond to C–O stretching from alcohol, carboxylic acid, or aldehyde groups, all due to functional groups of *A. vera* metabolites. In general, the IR spectrum of synthesized AgNPs repeats the structure of *A. vera* extract spectrum with some differences in the position and intensity of absorption bands. The absorption band corresponding to N–H bending vibrations at 1587 cm^−1^ is absent in the spectrum of green synthesized AgNPs, together with the disappearance of N-H stretching vibrations peaks at 2860 cm^−1^–2900 cm^−1^. It may be inferred that the protein compound was the probable reducing agent involved in synthesizing AgNPs. The purified nanoparticles did not exhibit absorption peaks at 1732 cm^−1^ due to C=O functional group. Peaks from 700 cm^−1^ to 900 cm^−1^ on extract spectra are attributed to aromatic groups. From the analysis of FTIR studies, it was confirmed that AgNPs were probably stabilized by bioactive components from *A. vera* extract (Figure-1), which might have formed a layer on the AgNPs (biological capping) that prevented agglomeration.

**Table-1:** MIC and MFC of phytofabricated silver nanoparticles using *Aloe vera* extract against *C. albicans*.

Fungal strains	Strain	MIC (µg/mL)	MFC (µg/mL)	MFC/MIC
*C. albicans*	C1	32	32	1
	C2	16	32	2
	C3	16	16	1
	C4	32	64	2
*C. albicans ATCC 10231*		4	8	2

MIC=Minimum inhibitory concentrations, MFC=Minimum fungicidal concentrations, *C. albicans*=*Candida albicans*

**Table-2:** Elemental composition of *A. vera* extract and AgNP phytoproduct.

Chemical elements	*A. vera* extract		AgNPs solution	
	
	Average fluorescence intensity, imp/µA	Standard deviation	Average fluorescence intensity, imp/µA	Standard deviation
Si	0.0617	0.0031	0.0624	0.0046
S	0.1335	0.0019	-	-
Cl	0.0274	0.0026	0.0163	0.0006
K	0.2459	0.0033	0.0869	0.0106
Br	0.0492	0.0023	-	-
Ag	-	-	0.8046	0.0305

*A. vera=Aloe vera*, AgNPs=Silver nanoparticles, Si=Silicon, S=Sulfur, Cl=Chlorine, K=Potassium, Br=Bromine, Ag=Silver

### Antifungal activity

**Figure-8:**
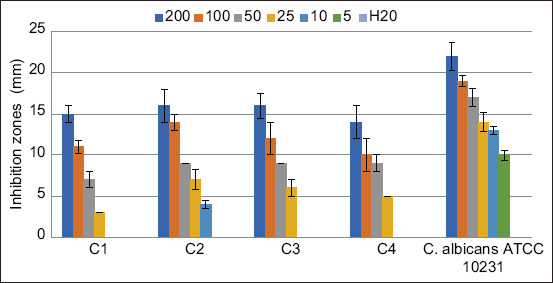
Antifungal activity of silver nanoparticles synthesized using *Aloe vera* extract obtained by well diffusion test. Six different concentrations were tested (200, 100, 50, 25, 10, and 5 μg/mL) and inhibition zones were presented as the average of three independent tests.

The antifungal activity of AgNPs synthesized using *A. vera* extract was tested against four clinical strains of *C. albicans* (C1, C2, C3, and C4) and a reference strain *C. albicans* ATCC 10231. Different concentrations of AgNPs were used (200, 100, 50, 25, 10, and 5 μg/mL). The results of the inhibition zones of the AgNPs at different concentrations are demonstrated in Figure-8. As expected, the negative control (distilled water used to prepare the solutions of AgNPs) showed no inhibition zone (0 mm) against all the fungi tested. The inhibition zones of AgNPs ranged from 10 mm to 22 mm against *C. albicans* ATCC 10231, 0 mm to 15 mm against C1, 0 mm to 16 mm against C2 and C3, and from 0 mm to 14 mm against C4. Regardless of the fungal strains, we observed that the inhibition diameters decreased with a decrease in the concentration of the AgNPs, which may indicate that the synthesized AgNPs are dose-dependent. Similar results are usually reported in most studies investigating the antimicrobial properties of nanoparticles and other drugs [2, 24, 34–37]. At lower amounts of AgNPs (5 μg/mL), all the clinical strains (C1, C2, C3, and C4) were not sensitive (0 mm), while at the same concentration, an inhibition zone of 10 mm was found against the reference strains *C. albicans* ATCC 10231. The result is not shown here, but it is essential to point out that similarly, *A. vera* extract itself showed no antifungal activity against C1, C2, C3, and C4, whereas a very weak antifungal activity (7 mm) was observed against *C. albicans* ATCC 10231. This observation may indicate that the clinical strains are phenotypically more resistant than the reference one, probably because they are passed through hostile conditions, leading them to better adaption to antifungal substances [24]. Although the mechanism of the action of nanoparticles is not yet fully elucidated [38], it can be hypothesized that clinical strains of *C. albicans* have phenotypically more chance of survival than non-resistant fungi when treated with antifungals. Moreover, it is well known that the well or disk diffusion tests are used as the preliminary study in screening the antimicrobial activity of an antimicrobial agent [39]; therefore, further investigation in determining the antifungal activity of AgNPs using MIC and MFC was needed. As shown in Table-1, the MIC values of AgNPs against *C. albicans* strains ranged from 16 μg/mL to 32 μg/mL against the clinical isolates (C1, C2, C3, and C4) and were 4 μg/mL against *C. albicans* ATCC 10231. Similarly, MFC, the lowest concentration of AgNPs, which totally kills the fungi, ranged from 8 μg/mL to 64 μg/mL for all *C. albicans*. Given the MBC/MIC ratios, it became apparent to conclude that AgNPs synthesized using *A. vera* extract can be considered as fungicidal agents since the MFC/MIC ratio was lower or equal to 4 (MBC/MIC ≤4) in all strains [40, 41]. Some studies have evaluated the antifungal activity of AgNPs, but a few existing studies have shown significant antifungal results similar to this study [1, 42]. The *in vitro* test performed in this study revealed that AgNPs synthesized in an eco-friendly manner present exciting antifungal activity and can be helpful in safety and environmental issues involving *C. albicans*. However, additional studies are needed to remove gray areas on toxicity, antimicrobial mechanism, *in vivo* antimicrobial activity, and pre-clinical and clinical trials before the AgNPs can be recommended for antimicrobial therapy.

## Conclusion

This study showed that AgNPs were successfully synthesized using *A. vera* extract. The average size of these phytofabricated nanostructures was 80.31 ± 10.03 nm. As confirmed by UV–visible spectrophotometry, PCCS, XRD analysis, energy-dispersive XRF spectrometry, and FTIR spectroscopy, the use of *A. vera* extract for the phytofabrication of AgNPs is fast, simple, and environmentally friendly. The synthesized AgNPs showed excellent antifungal activity against all the tested *C. albicans*. Further studies are underway to identify the phytochemicals involved in the bioreduction, capping, and stabilizing process.

## Authors’ Contributions

MMJA and PIV: Contributed to the study conception and design. MMJA, AKLD, MEY, MA, and MM: Performed the experiments. MMJA, MA, and MM: Contributed to sample collection and preparation. MMJA, MA, and MM: Analyzed the data. MMJA: Performed the statistical analysis. SAN, MN, AAG, KAA, YNV, ZAV, OS, DM, MR, and MMJA: Drafted and revised the manuscript. All authors have read and approved the final manuscript.
